# Functional Connectivity Analysis of NIRS Data under Rubber Hand Illusion to Find a Biomarker of Sense of Ownership

**DOI:** 10.1155/2016/6726238

**Published:** 2016-06-20

**Authors:** Naoki Arizono, Yuji Ohmura, Shiro Yano, Toshiyuki Kondo

**Affiliations:** ^1^Department of Computer and Information Sciences, Graduate School of Engineering, Tokyo University of Agriculture and Technology, 2-24-16 Naka-cho, Koganei, Tokyo 184-8588, Japan; ^2^School of Nursing and Rehabilitation Sciences at Odawara, International University of Health and Welfare, 1-2-25 Shiroyama, Odawara-shi, Kanagawa 250-8588, Japan

## Abstract

The self-identification, which is called sense of ownership, has been researched through methodology of rubber hand illusion (RHI) because of its simple setup. Although studies with neuroimaging technique, such as fMRI, revealed that several brain areas are associated with the sense of ownership, near-infrared spectroscopy (NIRS) has not yet been utilized. Here we introduced an automated setup to induce RHI, measured the brain activity during the RHI with NIRS, and analyzed the functional connectivity so as to understand dynamical brain relationship regarding the sense of ownership. The connectivity was evaluated by multivariate Granger causality. In this experiment, the peaks of oxy-Hb on right frontal and right motor related areas during the illusion were significantly higher compared with those during the nonillusion. Furthermore, by analyzing the NIRS recordings, we found a reliable connectivity from the frontal to the motor related areas during the illusion. This finding suggests that frontal cortex and motor related areas communicate with each other when the sense of ownership is induced. The result suggests that the sense of ownership is related to neural mechanism underlying human motor control, and it would be determining whether motor learning (i.e., neural plasticity) will occur. Thus RHI with the functional connectivity analysis will become an appropriate biomarker for neurorehabilitation.

## 1. Introduction

How does our brain distinguish our body from external objects? This question came up from the syndrome of somatoparaphrenia where patients cannot recognize their own body parts because of certain brain damage [1]. It is reported that this syndrome may be due to deficits of multisensory integration [2]. Therefore the self-identification, especially called as the sense of ownership, has been researched over the long time in multisensory research. However it is difficult to investigate the body ownership simply because all multimodal cues normally are bounded and not independent. For instance, if you touch an object, the touch feeling always follows the view of the touch and they come from the same body. In contrast, sensory cues on external objects are independent. Therefore it is possible to investigate the sense of ownership when stimuli are applied to the real body and fake counterparts [3]. This is why body ownership illusion where someone experiences external objects as one's own body parts has been intensively conducted; the methodology of rubber hand illusion (RHI) [4] has been extensively used. In RHI, participant is asked to watch a fake hand placed in front of him or her, while their real hand is hidden from the view. Then synchronous stimuli to the participant's hand and dummy counterpart lead the participant to feel the fake one as his or her hand. In conventional RHI studies, they suggest that integration between multimodal information such as visual, tactile, and proprioceptive information is important to provide the sense of ownership [5, 6].

Due to the simple setup, the mechanism of the sense of ownership has been researched through this methodology with neuroimaging like fMRI, PET, or EEG. Using fMRI, activity in prefrontal cortex [7], ventral premotor cortex [7–10], intraparietal sulcus [8, 11], and extrastriate body area [12] associated with the experience of the sense of ownership has been found. As for PET, it is reported that the body ownership is related to activity in the right posterior insula, sensorimotor cortex [13]. Besides, the studies using EEG show that the gamma band activity in the parietal area reflects the sense of ownership [14]. An enhanced somatosensory N140 component and ERP modulations have also been observed after the period of synchronous stimuli [15]. Moreover, TMS as well as neuroimaging methods have been used to investigate the brain activity during the illusion, which shows that significant activity in right temporoparietal junction and extrastriate body areas during RHI [16, 17]. The regions of activity associated with the sense of ownership are differently reported depending on neuroimaging methods. To summarize, it is at least obvious that wide brain areas including prefrontal cortex, ventral premotor cortex, sensorimotor cortex, parietal cortex, intraparietal cortex, and extrastriate body area are associated with the sense of ownership and these areas reflect multisensory integration.

Many neuroimaging experiments have conducted recently as mentioned above. Nevertheless, the studies investigating body ownership using near-infrared spectroscopy (NIRS) are yet to be studied. NIRS is also a noninvasive neuroimaging method. It measures the relative changes in oxygenated hemoglobin (oxy-Hb) and deoxygenated hemoglobin (deoxy-Hb) concentration. The reason why the studies on NIRS are limited is that NIRS has low spatial resolution in contrast to fMRI [18]. However, NIRS is easy to use and relatively robust against body sway because it can be very portable and is less invasive than fMRI and PET. Therefore participant's movement is nearly natural in measurement on NIRS, which is difficult to be achieved with other neuroimaging methods. Moreover, electromagnetic devices do not affect NIRS unlike fMRI, so we can introduce mechanical devices easily into RHI design for purpose of automatic setup. For these reasons, we measure brain activity during RHI using NIRS. In this experiment, we introduce automated setup for RHI because brain areas associated with the sense of ownership may respond to the observation of touch applied by another person [19]. We aim to eliminate the interference with multimodal integration. Then we induced RHI to participants with the stimulator and investigated whether the RHI using NIRS could show the activity associated with the sense of ownership.

We also analyze the functional connectivity of NIRS data during RHI, since dynamic relationship among brain areas associated with the sense of ownership has not yet been demonstrated well. Although there are several functional connectivity studies related to body ownership [14, 20], the studies focusing on causality are limited. Causality between different brain regions is more important and meaningful than simple correlation because neurons in brain transmit impulse as information to other neurons. One neuron must essentially cause another neuron. Therefore we calculate Granger causality between any two brain areas during RHI and consider significant causality as functional connectivity.

## 2. Material and Methods

### 2.1. Subjects

Ten healthy volunteers (8 males and 2 females; aged 20–24 years) participated in the following experiment. Eight out of the 10 subjects were right-handed and the rest were left-handed. None of the subjects had a history of psychiatric or neurological disorders. Vision was normal or corrected-to-normal. All subjects understood the purpose of the experiment and then provided written informed consent prior to participation. The study was approved by the local ethical committee at the Tokyo University of Agriculture and Technology in accordance with the Declaration of Helsinki.

### 2.2. Stimulator

For automating setup, there are several methodologies to avoid interferences in sense of ownership through observation of being touched by another person. Due to the development of virtual reality (VR) technique, VR-based RHI has been recently conducted. However, RHI strongly occurs in reality than in VR environments [21]. Thus, we developed and introduced an automating stroke of brushes for RHI.

**Figure 1:**
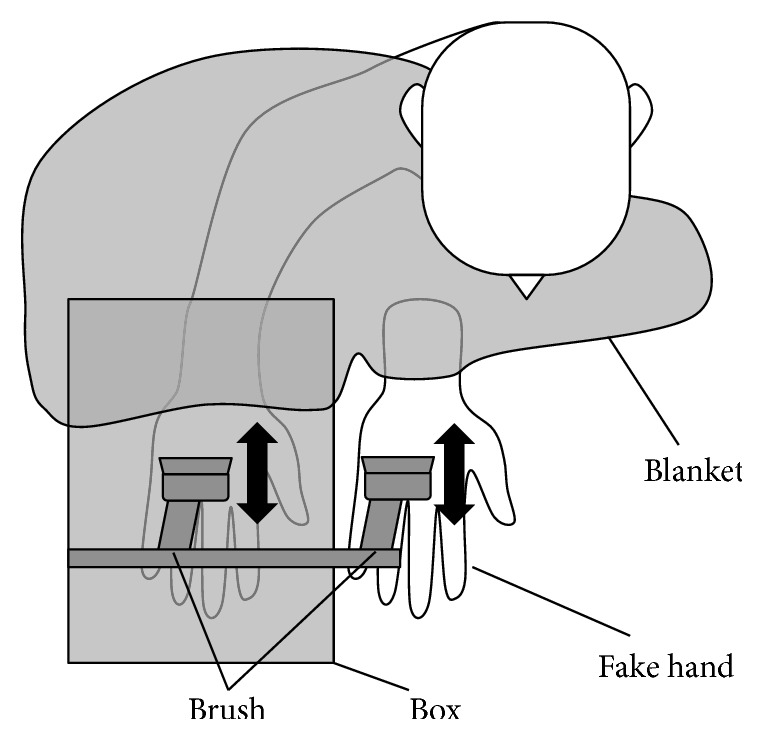
The setup used to induce RHI by automated brush stroking. The participant's hand and the fake hand were veiled by a box and both of hands wore cotton gloves which reduce the difference of shape and texture. Moreover a blanket covered his or her arm so that the participant watches only the fake hand.

Firstly, we attached two paintbrushes to servomotors where the speed of rotation and direction were controlled with a microcontroller (see Figure 1). The two brushes moved forward or backward. We were careful to regulate the maximum speed to reduce mechanical noise, which may disturb the illusion. The participant's right hand and the fake hand, which were veiled by a partition, wore cotton gloves in order to reduce the difference of shape and texture. The torque of the servomotor is 2.5 kgf·cm, which is enough to move the brushes which stimulate strongly the hand even over the cotton gloves. Moreover we put a board under their hands to adjust the height where each participant feels the stimulus on the back of their hand before beginning each session. A blanket covered their right arm so that participant watched only the fake hand. Moreover, both hands were aligned to the same direction (i.e., congruent) and were placed with 19 cm apart from each other. This is because hand ownership decreases when the fake hand is not aligned with the participant's hand or when an object instead of the fake hand is stroked [6].

### 2.3. Experimental Design

In the experiment, there were three experimental conditions (Synchronous, Asynchronous, Rest) on RHI. We manipulated the timing of the stroke stimuli on the both of hands, namely, (A) synchronous, two brushes stimulated the participant's hand and the fake hand simultaneously for 600 ms at intervals of 500 ms; (B) asynchronous, alternately the fake hand and the participant's hand were stimulated one by one for 600 ms at intervals of 500 ms; (C) rest, the two brushes did not stroke both hands at all. The duration of the stroke was determined by pilot study. These three conditions were repeated four times in random order to avoid context effect. Each condition lasted for 120 s, and we defined the unit as “session.” The participants took a rest for 90 s between each session. Besides they were asked to answer questionnaire on a 7-point Likert scale ranging from 1 (“completely disagree”) to 7 (“completely agree”) for 60 s after the end of both synchronous and asynchronous conditions to assess the hand ownership. This questionnaire comprised five statements: (Q1) “I felt as if the rubber hand was my hand,” (Q2) “Touch feeling is located on rubber hand,” (Q3) “I felt as if my real hand was turning rubbery,” (Q4) “It seems that I have more than one right hand,” and (Q5) “It seems that I have no right hand.” The first two statements (Q1) and (Q2) were relevant to the hand ownership. On the other hand, the other statements (Q3)–(Q5) served as suggestibility and task-compliance. All scores of each statement among all participants were tested by analysis of variance (ANOVA) and Tukey-Kramer post hoc comparison.

### 2.4. NIRS Data Acquisition

**Figure 2:**
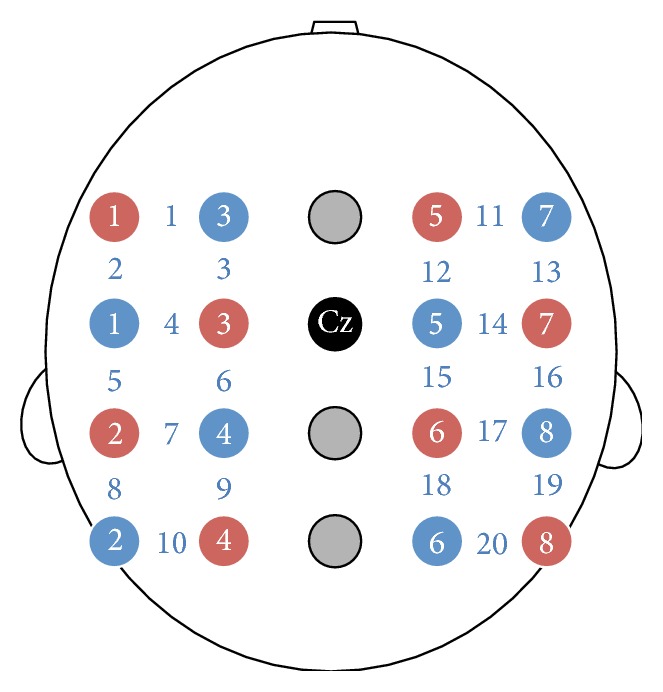
Configuration of NIRS channels (blue numbers) on the frontal-to-parietal areas. The eight pairs of emitting (red circles) and detecting probes (blue circles) were placed with a distance of 30 mm between the neighboring probes on the holder which was configured with a 4 × 5 array around Cz in international 10–20 system. 20 channels covered from frontal cortex to parietal cortex.

We used NIRS system (FOIRE-3000; Shimadzu, Kyoto, Japan) to record brain activity with 130 ms time resolution. FOIRE-3000 operates at 780, 805, and 830 nm wavelengths so as to measure relative concentration levels in oxy-Hb and deoxy-Hb by applying the modified Beer-Lambert law. We normalized the NIRS values at the beginning of each session to a baseline. The eight pairs of emitting and detecting probes were placed on the frontal-to-parietal areas using a holder which was configured with a 4 × 5 array around Cz in international 10–20 system (see Figure 2). 20 channels from frontal cortex to parietal cortex consisted of the emitting and detecting probes with a distance of 30 mm between the neighboring probes.

### 2.5. Analysis

For preprocessing, band-pass filter is used commonly to remove some noise in NIRS data because the noise and a slow drift can be induced by change of posture and arousal level, fatigue, continuing warming, and so on [22]. We applied 3rd order Butterworth band-pass filter of 0.009–0.1 Hz to eliminate the drift component and physiological noise such as respiratory, cardiac, and motion artifacts and flatten the baseline [23]. Then we set the beginning part of NIRS data in each session as the baseline, as we mentioned above. This is because we took a longer rest (30 s) between each session.

For statistical analysis, significant activation at all channels during each session was established using *t*-test across spatial domains. Namely, we calculated peak NIRS value at each channel across time domains of each session, and then we classified these sessions into two sets and defined illusion set and nonillusion set as follows: illusion set (answered (Q1) + (Q2) ≥ 10 in synchronous condition) and nonillusion set (answered (Q1) + (Q2) ≤ 6 in asynchronous condition). This is because (Q1) and (Q2) were relevant to the extent of RHI and were useful to see the brain activity more influenced by the illusion. After that, an unpaired *t*-test assessed whether peak NIRS values were different between illusion and nonillusion sets so that we made unpaired comparison analysis not between subjects but between illusory and nonillusory conditions.

Furthermore, we evaluated functional connectivity between arbitrary two channels using Granger causality [24]. Granger causality is statistical method to investigate causal relations among simultaneously acquired signals, which is based on linear prediction theory of time series. The concept of Granger causality is simple as follows: assume that there are two stationary variables *x*_1_ and *x*_2_. In a *p*th order vector autoregressive formulation, and a univariate autoregressive formulation, the *x*_1_ component takes each form

(1) x1t=∑j=1pA1,jx1t−j+∑j=1pA2,jx2t−j+εt,x1t=∑j=1pA1,j′x1t−j+ε′t,

where *ε* corresponds to the residual, which is independent and identically distributed (iid). There is no dependence of *x*_1_ on *x*_2_ if *A*_2_ = 0. In contrast, *x*_2_ causes *x*_1_ if the coefficients in *A*_2_ are significantly different from 0. In other words, if the covariance of *ε* is smaller than *ε*′ by the inclusion of *x*_2_, then we can say *x*_2_ has causality on *x*_1_. Therefore, it can be tested by performing an *F*-test of the difference between covariance of *ε* and covariance of *ε*′

(2) $$\begin{array}{c} F_{x_{2}\to x_{1}}=\ln\frac{cov\left(\varepsilon^{\prime }\right)}{cov\left(\varepsilon \right)}. \end{array}$$

The appropriate order of the model *p* was determined by Bayesian information criteria avoiding overfitting a finite data sequence. Besides, coefficients *A* were estimated by ordinary least squares to minimize the model error. In the study, we used multivariate Granger causality analysis. Bivariate Granger causality may lead to ambiguous results due to mediated causal influences. For instance, if there is no direct causal influence *x*_2_ → *x*_1_, although there are lagged dependencies of *x*_1_ and *x*_2_ on *x*_3_ (i.e., *x*_2_ → *x*_3_, afterwards *x*_3_ → *x*_1_), bivariate analysis would infer spurious causality *x*_2_ → *x*_1_. We used multivariate analysis because we recorded 20 time series and all other observations *x*_3_ ⋯ *x*_*n*_ may give misleading causality. We may reconsider two formulas as

(3) x1t=∑j=1pA1,jx1t−j+∑j=1pA2,jx2t−j+∑j=1p∑k=3nAk,jxkt−j+εt,x1t=∑j=1pA1,j′x1t−j+∑j=1p∑k=3nAk,j′xkt−j+ε′t

with the conditioning other observations *x*_3_ ⋯ *x*_*n*_ included in both regressions. The causality *x*_2_ to *x*_1_ conditioned on *x*_3_ ⋯ *x*_*n*_ is defined again as

(4) $$\begin{array}{c} F_{x_{2}\to x_{1}\;|\;x_{3}\cdots x_{n}}=\ln\frac{cov\left(\varepsilon^{\prime }\right)}{cov\left(\varepsilon \right)}. \end{array}$$

We calculated multivariate Granger causality above using MVGC toolbox [25] and averaged out the number of connectivity for illusion set and nonillusion set. Chi-square test for independence was performed to see the difference of the total connectivity between these sets.

In the study, we utilized 120-s long NIRS data for the above-mentioned analysis (we dubbed them as original data). However, we could not have confidence when the illusion exactly occurred, because we did not instruct the participants to give a response once they experienced the illusion. Another RHI study reported that the illusion started after 14.3 ± 9.1 s (mean ± SD) [26]. Thus we trimmed 30 s off from the beginning of each trial and exploited the 90-s NIRS data as modified data. The modified data would be considered that it does not include the time before the onset of the illusion. Then we analyzed both original (120-s) and modified (90-s) data for functional connectivity.

## 3. Results

### 3.1. Questionnaire

**Figure 3:**
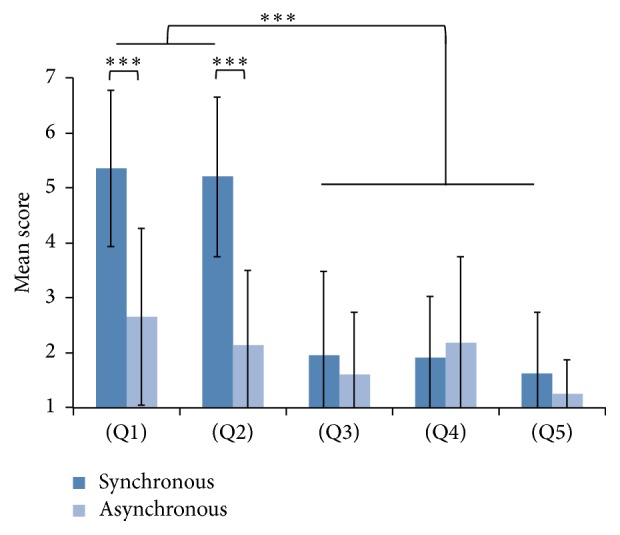
Mean scores of questionnaire which 10 participants answered. The questionnaire comprised two illusory statements (Q1, Q2) and three control statements (Q3, Q4, Q5). Error bars denote standard deviation. They reported that they experienced RHI in synchronous condition rather than in asynchronous condition. They also disagreed with all of control statements contrasted to illusory statements in synchronous condition. ^*∗∗∗*^ *p* < .001.

The completed questionnaire shows that most of the participants experienced RHI in synchronous condition (see Figure 3). The mean of rating score for (Q1) “I felt as if the rubber hand was my hand” across all participants in synchronous condition was 5.4 (S.D. 1.4) and that for (Q2) “Touch feeling is located on rubber hand” during synchronous condition was 5.2 (S.D. 1.4). Moreover, those for (Q1) and (Q2) in asynchronous condition were 2.7 (S.D. 1.6) and 2.2 (S.D. 1.4) for each. Therefore the questions capturing the experience of RHI were rated between 5 and 7, which means confirming the presence of RHI during synchronous condition, and the mean scores of (Q1) and (Q2) were significantly higher in synchronous condition than in asynchronous condition (both *p* < .001, Wilcoxon signed-rank test). The difference of rating between the illusion questions (i.e., Q1, Q2) and the other control questions (Q3, Q4, and Q5) was significant (*F*(4,195) = 77.94, *p* < .001, ANOVA; contrast comparing (Q1, Q2) to (Q3, Q4, Q5), all *p* < .001, Tukey-Kramer post hoc comparison).

### 3.2. NIRS

**Figure 4:**
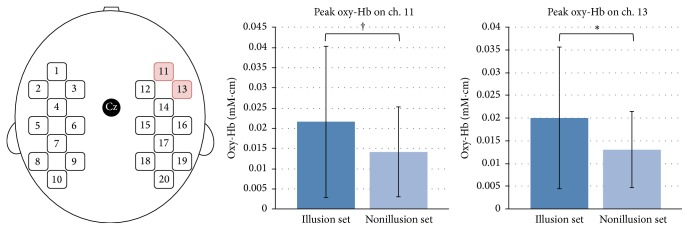
Significant activation of illusion set compared with nonillusion set. The activation of oxy-Hb on ch. 13 (right motor related areas) was significantly different between two sets. In addition, the activation of oxy-Hb on ch. 11 (right frontal cortex) in illusion set was also slightly higher than nonillusion set. ^*∗*^ *p* < .05 and ^†^ *p* < .10.

From the result of questionnaire, we classified the sessions into illusion set and nonillusion set (see Analysis and Table 1) and calculated peak NIRS values of oxy-Hb on each channel during task for illusion set and nonillusion set (see Analysis and Figure 4). A *t*-test analysis showed there was significant difference between two sets on ch. 13 (*t*-value = 2.15, df = 59, *p* = .036, unpaired two-tailed *t*-test). The activation on ch. 11 of oxy-Hb in illusion set was slightly higher than that in nonillusion set (*t*-value = 1.86, df = 59, *p* = .068, unpaired two-tailed *t*-test), although the other channels did not show significant activation (all *p* > .10, unpaired two-tailed *t*-test).

### 3.3. Multivariate Granger Causality

**Figure 5:**
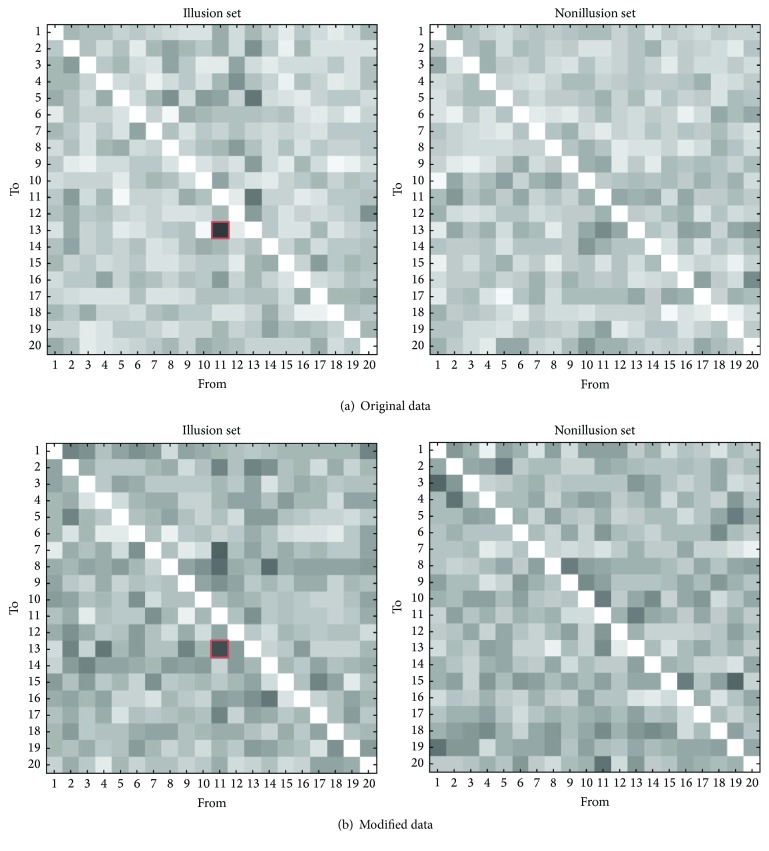
Average functional connectivity matrix. Each square represents the rate of connectivity based on oxy-Hb. The thicker color of square, the more connectivity was found. (a) The matrices got by analyzing original data. The rate of connectivity from ch. 11 (right frontal cortex) to ch. 13 (right motor related areas) in illusion set (left matrix, red square) was 73.3%, which was the most among all connectivity and significantly more than that in nonillusion set. (b) The matrices got by modified data. The rate of connectivity from ch. 11 to ch. 13 in illusion set (left matrix, red square) was 66.7%. The rate was also the most among all connectivity, although it was not significantly but slightly more than that in nonillusion set.

Finally, we averaged the number of functional connectivity for the illusion set and nonillusion set in original and modified data (see Analysis and Figure 5). In the original data, the rate of connectivity from ch. 11 to ch. 13 in illusion set was 73.3%, which was the highest among all connectivity. Chi-square test showed that the number of the connectivity was significantly higher than that in nonillusion set (*χ* ^2^ = 3.98, df = 1, *p* = .046, chi-square test). Meanwhile, the rate of connectivity from ch. 11 to ch. 13 in illusion set in modified data was 66.7%, which was also the highest among all connectivity. However, chi-square test showed that the number of the connectivity was not significantly but slightly more than that in nonillusion set (*χ* ^2^ = 2.86, df = 1, *p* = .091, chi-square test).

## 4. Discussion

In the study, we developed automatic stimulator for RHI, which could eliminate the interference of human interaction. Human and social interaction like the observation of touch by another person leads to the activation on premotor cortex, primary somatosensory cortex, and extrastriate body area [19]. Therefore, the automatic system for RHI is valuable to see the pure activation associated with the sense of ownership. Moreover, we can manage the speed and timing of stimuli. It is difficult for humans to always stimulate both of hands at the exact same time. A study suggests that discrepancy of more than 300 ms between visual stimulation of the rubber hand and tactile stimulation to the participant's own hand will weaken RHI [27]. To induce strong RHI, the time of visuotactile stimuli should match simultaneously for multisensory integration. The quality of stimuli also should be consistent between participants and sessions. By such reasons, the precise control of stimulus by the automatic stimulator provided stability and reproducibility of illusion. In particular, we developed unmediated stimulator, not with virtual reality because the unmediated condition produced stronger illusion [21]. However, it was recently possible to make the participant experience the strong illusion in immersive virtual reality (IVR) where the participant's body is replaced with a life-sized virtual body seen from first person perspective [28]. It is expected that the parameters, which we defined in the study, will be useful to set up the illusion in IVR that is enough to represent the participant's body.

The results of the questionnaire showed that most of participants felt the illusion. The mean score of (Q1) and (Q2), which are relevant to RHI, was significantly higher than that of (Q3), (Q4), and (Q5), which was used as control. In addition, the mean score of (Q1) and (Q2) was significantly higher in synchronous condition than in asynchronous condition. RHI is induced by synchronous stimuli, not asynchrony [4], because the visuotactile information should be integrated at the same time. Therefore, the automatic stimulator we developed could provide RHI to most of participants, and their brains should be activated rather during the illusion than the nonillusion.

In fact, we could find the activation in specific brain areas during the present illusion contrasted to the absent illusion. The activation on the right motor related areas was found in rather illusion set than nonillusion set. Many studies reported the involvement of ventral premotor cortex during RHI [7–10]. They proposed that ventral premotor cortex seems to be crucial in multisensory integration. In a macaque study, premotor cortex actually is area of convergence of visual, tactile, and proprioceptive information [29]. Their studies are consistent in a role of ventral premotor cortex. Our results also fit with these results where motor related areas are associated with multisensory integration, namely, the sense of ownership. In addition to the motor related areas, we also found the activation on the right frontal cortex. Prefrontal cortex is also correlated with the sense of ownership and self-location [7, 30]. Many studies suggest that prefrontal cortex plays a role in self-referential processing [31] and collection of multisensory information and control of action by giving and taking this information with motor areas [32]. Furthermore, it is reported that damage to prefrontal cortex causes abnormal bodily self-consciousness [33]. For these evidences, we identified the areas that are associated with the sense of ownership in the study of RHI using NIRS.

In analysis of Granger causality, we investigated the functional connectivity in the data starting at 30 s, in addition to the analysis in the original data [34, 35]. Although the results were a little different between the original and modified data, we still found significant functional connectivity from the frontal cortex to the motor related areas where the activation has been identified. As mentioned above, prefrontal cortex collects visuotactile information and exchanges it with premotor cortex. The results suggest that frontal cortex and motor related areas communicate with each other when the sense of ownership changes. It also indicates that motor related areas integrate visuotactile information for reconstructing the sense of ownership as other studies revealed. The exchange of the information between prefrontal cortex and premotor cortex reflects the selection of the action to execute [32]. Therefore, there is a possibility that RHI helps participants to activate specific brain areas that play a role of action selection. Other studies actually reveal that the brain mechanisms of body ownership overlap the mechanisms of motor imagery that has been used to study action selection [36]. Our results also follow the suggestion that the sense of ownership is related to neural mechanism underlying motor control. Note that the activation found in this study was in the right hemisphere, although participants' right hands were stimulated. Other studies reported the predominant involvement of the right inferior frontoparietal network when people recognize the changes of their body representation [37, 38]. The study also suggests that right frontal and motor areas are correlated with the sense of ownership.

NIRS has some advantages in measuring the sense of ownership. Most of the experiments assessing the sense of ownership require participants to have natural posture. Namely, participants need to stand up in the experiments involving whole body. NIRS imposes less constraint on participants compared with other neuroimaging methods such as fMRI. Therefore, NIRS study would deal with the experiment of the sense of ownership, which other neuroimaging could not. Moreover, NIRS is used to detect mental disorders (e.g., depression, bipolar disorder, and schizophrenia) by comparing the hemodynamics during task and rest. In particular, schizophrenia patients feel stronger RHI contrasted to healthy people [39, 40]. It is expected that the Granger causality analysis using NIRS during RHI will be used as biomarker for the diagnosis of mental disorders or neurorehabilitation for paralyzed patients.

However, NIRS has low spatial resolution contrasted to fMRI and EEG. Consequently, from the results, we have not observed the activation on sensorimotor cortex and parietal cortex in the illusion, which should appear. Furthermore, we identified some inexplicable functional connectivities where the brain regions were not correlated with the sense of ownership. It would be necessary to clarify some inexplicable connectivities introducing regularization such as Lasso that enhances the prediction accuracy in regression analysis. Therefore, a specific marker that indicates if the functional connectivity is meaningful is expected. Further investigation is required for identifying individual variability of the NIRS values and the connectivity.

## 5. Conclusions

In the study, we measured the brain activity during RHI induced by the automated stimulator with NIRS and investigated the significant activation and functional connectivity by Granger causality. As a result, we found a reliable activity and connectivity from the frontal to the motor related areas during the illusion. Therefore, there is a possibility that frontal cortex and right motor related areas communicate with each other when sense of ownership is modified. This exchange of the information between frontal cortex and motor related areas reflects the selection of the action to execute. Our results suggest that sense of ownership is associated with neural mechanism underlying motor control. It is expected that the Granger causality analysis with NIRS during RHI will be used as a biomarker for diagnosis of mental disorders or neurorehabilitation for paralyzed patients. However, we also found some inexplicable connectivities in the results. Further investigation of the functional connectivity is required to remove some connectivities as artifact by introducing the regression analysis with regularization.

## Figures and Tables

**Table 1:** The number of sessions in illusion and nonillusion set. “I” means that the participant answered (Q1) + (Q2) ≥ 10 after the session in synchronous condition, and “N” means that the participant answered (Q1) + (Q2) ≤ 6 in asynchronous condition. We got 30 sessions where participants experienced the illusion and 31 sessions where participants did not experience the illusion at all and defined those sessions as illusion set and nonillusion set.

	Sync				Async			
Subject 1	I	I	I	I	N	N	N	N
Subject 2	—	—	—	—	N	N	N	—
Subject 3	I	I	I	I	N	N	N	N
Subject 4	I	—	I	—	N	N	N	N
Subject 5	—	I	I	I	N	N	N	N
Subject 6	I	I	I	I	N	—	—	—
Subject 7	I	I	I	I	N	N	N	N
Subject 8	I	I	I	I	—	N	N	—
Subject 9	I	I	I	—	N	—	N	N
Subject 10	—	I	—	I	N	N	—	—

Total	30 sessions in illusion set				31 sessions in nonillusion set			
